# Distinct mutational signature and clonal evolution in constitutional mismatch repair deficiency-associated high-grade gliomas

**DOI:** 10.1016/j.isci.2026.115029

**Published:** 2026-02-14

**Authors:** Chang Li, E. Zeynep Erson-Omay, Yavuz Koksal, Ekrem Unal, Buket Kara, Kaya Bilguvar, Yahya Paksoy, Nimetullah Alper Durmus, Ali Kurtsoy, Huseyin Per, John Rosendahl Østergaard, Murat Günel, Ahmet Okay Çağlayan

**Affiliations:** 1 Department of Neurosurgery/Neuro-oncology, Sun Yat-sen University Cancer Center, State Key Laboratory of Oncology in South China, Sun Yat-sen University Cancer Center, Guangzhou 510060, P.R. China; 2 Departments of Neurosurgery, Yale School of Medicine, New Haven, CT 06510, USA; 3 Departments of Yale Program in Brain Tumor Research, Yale School of Medicine, New Haven, CT 06510, USA; 4 Departments of Pediatrics, Selcuk University School of Medicine, Konya 34848, Turkiye; 5 Pediatric Hematology and Oncology Clinic, Medical Point Hospital, Gaziantep 27584, Turkiye; 6 School of Health Sciences, Hasan Kalyoncu University, Gaziantep 27410, Turkiye; 7 Departments of Pediatrics, Erciyes University School of Medicine, Kayseri 38039, Turkiye; 8 Department of Medical Genetics, School of Medicine, Acibadem University, Istanbul 34684, Turkiye; 9 Departments of Radiology, Selcuk University School of Medicine, Konya 34848, Turkiye; 10 Departments of Neurosurgery, Erciyes University School of Medicine, Kayseri 38039, Turkiye; 11 Department of Pediatrics, Aarhus University Hospital, Skejby, Denmark; 12 Department of Medical Genetics, School of Medicine, Dokuz Eylul University, Izmir 35340, Turkiye; 13 Department of Molecular Medicine, Institute of Health Sciences, Dokuz Eylul University, Izmir 35340, Turkiye; 14 Rare and Undiagnosed Disease Platform, RAREBOOST Project. IBG-Izmir Biomedicine and Genome Center, Izmir 35340, Turkiye

**Keywords:** molecular biology, cancer

## Abstract

Constitutional mismatch repair deficiency (CMMRD) is a rare cancer-predisposing syndrome. Recent studies have advanced our understanding of the genomic and epigenomic features of this disease, however, the mutational signatures and clonal evolution of CMMRD-associated high-grade gliomas (HGGs) requires further investigation. Herein, we analyzed the mutational signature and clonal evolution of 25 CMMRD-associated HGGs. Germline biallelic mutations in *MSH6* (56.0%), *PMS2* (36.0%), *MLH1* (8.0%) were identified. Patients showed early onset (5.8 ± 4.2 years) and poor prognosis (progression-free survival 16 ± 18.0 months). Notably, we identified distinct mutational signatures, evolution pattern and clinical outcome between *MSH6* and *PMS2* subgroups, showing enriched SBS6 and SBSS21, respectively, which were found to correlate with prognosis. Clonal evolution model indicated early *POLE*/*POLD1* events and survival of founding clone during tumor recurrence. These findings provide valuable insights into the genomic landscape and clinical outcomes of CMMRD-associated HGGs, emphasizing the critical role of mutational signature and tumor evolution in tumorigenesis and patient prognosis.

## Highlights

• CMMRD-associated gliomas show distinct mutational and evolutionary profiles
• *MSH6* and *PMS2* subgroups show unique mutational signatures associated with prognosis
• Clonal evolution model shows early *POLE*/*POLD1* events and survival of founding clone

## Graphical abstract

**Figure:**
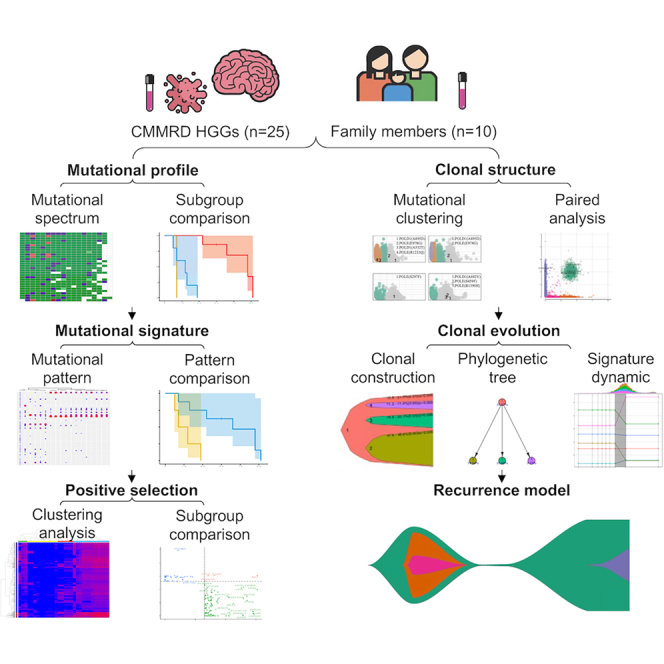

## Introduction

Constitutional mismatch repair deficiency (CMMRD) syndrome is a rare autosomal recessive inherited cancer predisposition syndrome. CMMRD is classically caused by biallelic germline mutations in one of the mismatch repair (MMR) genes including *MSH6*, *PMS2*, *MSH2*, and *MLH1*, and is characterized by early development of pediatric malignancies.^1^^,^^2^^,^^3^

CMMRD poses significant diagnostic challenges to clinicians despite the efforts of establishing diagnostic criteria and clinical guidelines in recent years.^4^^,^^5^ Diagnosis of CMMRD can be confirmed by DNA sequencing according to the diagnostic criteria proposed by the European consortium C4CMMRD, while immunohistochemistry has also been suggested as a highly sensitive diagnostic method.^6^^,^^7^ Screening of susceptible populations is thus crucial for the early diagnosis, and for this purpose, a scoring system based on a pleiotropic CMMRD phenotype has been proposed. However, these phenotypes are non-specific to CMMRD patients, causing difficulty of the diagnosis particularly in patients with malignant brain tumors.^2^^,^^5^^,^^6^ Family history of malignancy and consanguineous marriage can raise the suspicion of CMMRD, however, in many cases such history can be lacking.^8^^,^^9^^,^^10^^,^^11^^,^^12^ More recent recommendations from the international consensus working group include four definitive criteria and three likely diagnostic criteria.^13^ Nevertheless, confirmative diagnosis of CMMRD still depends crucially on the test of germline mutations in the MMR gene, as under the new consensus, ancillary testing and clinical phenotype are required besides germline MMR mutation testing, unless in trans biallelic pathogenic variants were confirmed.

Patients with CMMRD-associated HGGs have poor prognosis. Previous studies managed to summarize the clinical outcome of CMMRD HGG patients from limited clinical data and reported a 3-year OS of 20.5% and a median OS of under 15 months after standard treatment.^5^^,^^14^ Recent large-scale studies have advanced our understanding of the clinical and molecular characteristics of CMMRD-associated HGGs.^7^^,^^15^^,^^16^^,^^17^ The study by Negm et al. provided the first population-based assessment of the prevalence of primary mismatch repair deficiency (MMRD) in a large cohort of glioma patients aged 0–40 years^15^ Their findings indicated that HGGs with primary MMRD may be more common than previously estimated, with a reported prevalence ranging from 3.7% to 12.4% among young adults. Notably, the study identified particularly poor survival in primary MMRD patients with IDH-mutant HGGs. Ercan et al., investigated the clinical and molecular landscape of 339 malignancies in 201 CMMRD patients.^17^ They found that CNS tumors were the most frequent CMMRD-associated malignancies, characterized by early onset and poor overall survival. Among these, the majority were HGGs with 66% classified as glioblastomas and 14% as anaplastic astrocytoma. Standard therapeutic approaches can be ineffective, notably, the largely used temozolomide (TMZ) treatment in HGGs may not improve patient outcome due to the strong resistance of MMR-deficient cells to DNA alkylating agent.^18^^,^^19^ Immunotherapy is considered a promising approach given the high mutation burden of these tumors, however, its efficacy still needs to be validated. While immune checkpoint inhibitor (ICI) treatment has been reported to benefit the survival in certain genomic subgroups, poor survival and low response rate to PD-1 inhibitor have also been previously reported.^19^^,^^20^^,^^21^^,^^22^ High mutation burden and microsatellite indels have been suggested as potential predictors of ICI response, however, their roles tend to vary across genomic subgroups.^21^^,^^22^ Thus, the screening of therapeutic targets and prognostic biomarkers should be the focus of improving diagnosis and prognosis of CMMRD-associated HGGs, which requires comprehensive analysis and in-depth study of the molecular profile.

Previous studies investigating hypermutated tumors and MMR deficient malignancies have described the genomic profile of CMMRD-associated HGGs, however, studies that focused on the mutational signature and clonal architecture of CMMRD-associated HGGs are still lacking. In addition, our previous study has identified a distinct HGG subgroup with a favorable prognosis defined by *POLE* and ultra-hypermutation, suggesting distinct molecular signatures associated with the clinical outcomes of CMMRD-associated HGGs.^23^ Therefore, the underlying mutational process and the evolutionary trajectory is critical for classifying CMMRD-associated HGGs and urge further investigations.

In this study, we conducted comprehensive investigation of the genomic profile of CMMRD-associated HGGs to identify their mutational signature and clonal evolution. We summarized distinct signatures in MMR subgroups associated with prognosis, and revealed the evolutionary history of both primary and recurrent CMMRD-associated HGGs. We reasoned that our results should improve the understanding of the mutational process and tumor evolution pattern in CMMRD-associated HGGs. This study provides valuable insights into the clonal architecture and tumor evolution of CMMRD-associated HGGs, which may contribute to the growing body of research on this rare disease alongside other key studies in the field.

## Results

### Clinical characteristics and genomic landscape

We analyzed 25 CMMRD-associated HGGs, with whole exome sequencing (WES) performed on 18 samples, and next-generation sequencing based on a customed gene panel for 7 samples.^24^ Additionally, 10 blood samples from family members were included to track germline MMR mutations (Tables S1–S3). Germline homozygous or compound heterozygous MMR mutations including *MSH6* (*n* = 14, 56.0%), *PMS2* (*n* = 9, 36.0%), and *MLH1* (*n* = 2, 8.0%) were identified in all 25 CMMRD-associated HGGs. Patients exhibited early onset of HGG, with a median diagnosis age of 5.8 ± 4.2 (Median±SD) years and a progression-free survival (PFS) of 16 ± 18.0 months.

Table S1. Clinical characteristics of analyzed samples from 25 CMMRD-associated HGGs

Table S2. Genomic landscape of MMR and POLE/POLD1 mutations of HGG patients and CMMRD families

Table S3. Clinical features of the CMMRD-associated HGG patients

**Figure 1:**
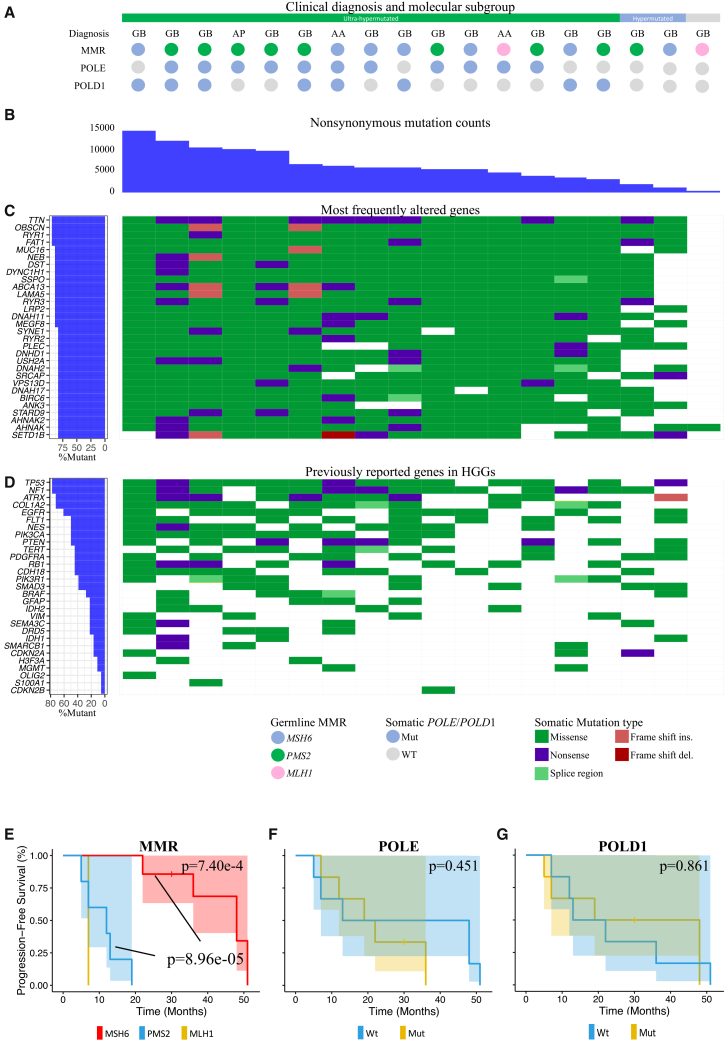
Clinical characteristics and genomic landscape of CMMRD-associated HGGs (A) Diagnosis, germline homozygous MMR and somatic POLE/POLD1 mutations in CMMRD-associated HGGs. (B) The number of nonsynonymous somatic mutations across all samples. (C) Somatic mutations in the most frequently altered genes (top 30) across the 18 samples and in (D) previously reported genes in HGGs. The left panel shows the mutation frequency of each gene. (E) Kaplan-Meier curves of progression free survival (PFS) in the CMMRD HGG patients with identified germline homozygous MMR mutations. The *p* value was calculated via the log rank test. (F) Kaplan-Meier curves of PFS comparing the POLE-wildtype and POLE-mutant cases. (G) Kaplan-Meier curves of PFS comparing the POLD1-wildtype and POLD1-mutant cases.

CNS cancers have been reported to harbor the highest TMBs among CMMRD tumors.^17^ We observed high tumor mutational burden (TMB) in CMMRD-associated HGGs, with an average TMB of 159.1 ± 170.2 Mut/Mb (Figure 1 and S1). Hypermutation (>10 Mut/Mb) or ultra-hypermutation (>100 Mut/Mb) as defined in the previously study was observed in most cases (94.4% and 17/18).^25^ Notably, tumors with somatic *POLD1* mutations had significantly higher TMB (310.6 ± 157.0 Mut/Mb, *p* = 1.68e-2). No significant differences in TMB among MMR subgroups were observed. The CNV burden of CMMRD HGGs was 437.6 ± 484.9 MB (S4 and S1).

Table S4. Allele-specific copy number analysis of CMMRD-associated HGG

Frequent somatic mutations in DNA polymerase ε (Pol ε and *POLE*) and polymerase δ (Pol δ and *POLD1*) were noted, particularly in ultra-hypermutated tumors (Figures 1A and S2). All ultra-hypermutated CMMRD-associated HGGs carried driver mutations in *POLE* (73.3% and 11/15) and/or *POLD1* (53.3% and 8/15), whereas the 2 hypermutated samples were *POLE*/*POLD1* wild type (S5). We observed a subset of *POLE* variants that have been previously reported in hypermutated tumors. For example, F104, P286, S297, P436, S459, and S461 are either conserved residues or important exonuclease motif in *POLE*, while E318 is in an ExoI motif in *POLD1*.^26^^,^^27^ *POLE* p.E978G affects the DNA polymerase domain and has been validated in pediatric HGGs with a high TMB.^16^

Table S5. Mutations in POLE/POLD1 in CMMRD HGGs

Additionally, we observed several rare mutations in *POLE* and *POLD1* in CMMRD-associated HGGs, which remained to be validated experimentally. *POLE* p.S964Y, p.R680C, and p.R847W affects in the polymerase domain, and S964 is highly conserved in *POLE*. These have not been observed at significant frequency in large population nor in *POLE*-related conditions, and were predicated to be deleterious.^28^ *POLE* c.285 + 6C>T hits the splice site and may affect the exonuclease domain. *POLD1* p. A692V and p.A915V located in the polymerase domain, and A692 was highly conserved in this gene. These two *POLD1* mutations were suggested to have a deleterious effect on the protein via in silico analysis, however, have not been observed in large population or reported with validated pathogenicity. While driver mutations mostly either affect the exonuclease and polymerase domains of the genes, the biological significance of Pol mutations outside these domains can be difficult to assess. These potential passenger mutations were found in 11 of the 18 CMMRD-associated HGGs that underwent WES and were excluded from further analysis.

WES analysis revealed the mutational spectrum of CMMRD-associated HGGs. Key frequently mutated genes included *TTN*, *OBSCN*, *RYR1*, and *FAT1* (94.4% and 17/18 of cases) (Figure 1C). Other notable mutations involved *MUC16*, *NEB*, *DST*, *DYNC1H1*, *SSPO*, *ABCA13*, *LAMA5*, *RYR3*, *LRP2*, *DNAH11*, and *MEGF8* (88.9% and 16/18 of cases). Mutations in previously reported HGG drivers including *TP53*, *NF1*, *ATRX*, *COL1A2*, *EGFR*, *FLT1*, *NES*, *PIK3CA*, *PTEN*, *TERT*, *PDGFRA*, and *RB1* can be observed in >40% of the cases (Figure 1D). *TP53* mutation was particularly enriched in CMMRD-associated HGGs (77.8% and 14/18), followed by *ATRX* mutations (72.2% and 13/18). Downregulation of p53 have been widely observed in glioblastoma, while *ATRX* mutations has been reported in nearly 1/3 of pediatric GBM and were associated with increased SNV mutation rates.^29^^,^^30^ Notably, mutations were frequently observed in genes participating RAS/PI3K/AKT signaling, including *NF1* (14/18), *PTEN* (9/18), *PIK3CA* (9/18), *PDGFRA* (8/18), and *PIK3R1* (7/18). Mutations in *RB1* (9/18), *EGFR* (10/18), *TERT* (8/18) were detected in around half of the cases. On the other hand, mutations in *CDKN2A*, *SMARCB1*, *IDH1*, *IDH2*, and *BRAF* were evident in around 20% cases. *MGMT* and *H3F3A* mutations were observed in only 2 cases while *CDKN2B* was observed only in 1 case.

Importantly, the PFS of CMMRD-associated HGG patients varied significantly among MMR subgroups (7.4E-04) (Figure 1E). HGGs harboring germline homozygous or compound heterozygous mutations in *MSH6* demonstrated significantly longer PFS than those harboring *PMS2* mutations (*p* = 9.0e-05). The prognosis of *MLH1* or *MSH2* HGGs was not analyzed in our study due to limited sample size. Notably, Ercan et al. reported poorer 5-year OS in patients with CNS tumors harboring *MLH1* or *MSH2* mutations compared to those with *PMS2* or *MSH6* mutations.^17^ While somatic *POLE* and *POLD1* mutations were frequently observed, we observed no significant differences between the mutant and the wild type subgroups (*p* = 0.451, *p* = 0.861, respectively). Notably, Negm et al. identified a distinct subgroup of IDH-mutant gliomas with primary MMRD, which were associated with poor prognosis.^15^ Similar cases were also observed in our cohort (*n* = 4, S1). However, we did not observe a significant difference in PFS between IDH-mutant and IDH-wildtype CMMRD-associated HGGs in our cohort, possibly due to the limited sample size (S3). A multivariate Cox regression analysis incorporating the MMR, *POLE*/*POLD1* and *IDH* status have been performed to exclude potential confounding factors. However, no significant association with PFS was observed (S6). The clinical significance of these findings needs to be further validated in large-scale studies.

Table S6. Multivariate Cox regression analysis of MMR, POLE/POLD1 and IDH status associated with progression-free survival (PFS)

### Mutational processes and patterns in CMMRD-associated HGGs

**Figure 2:**
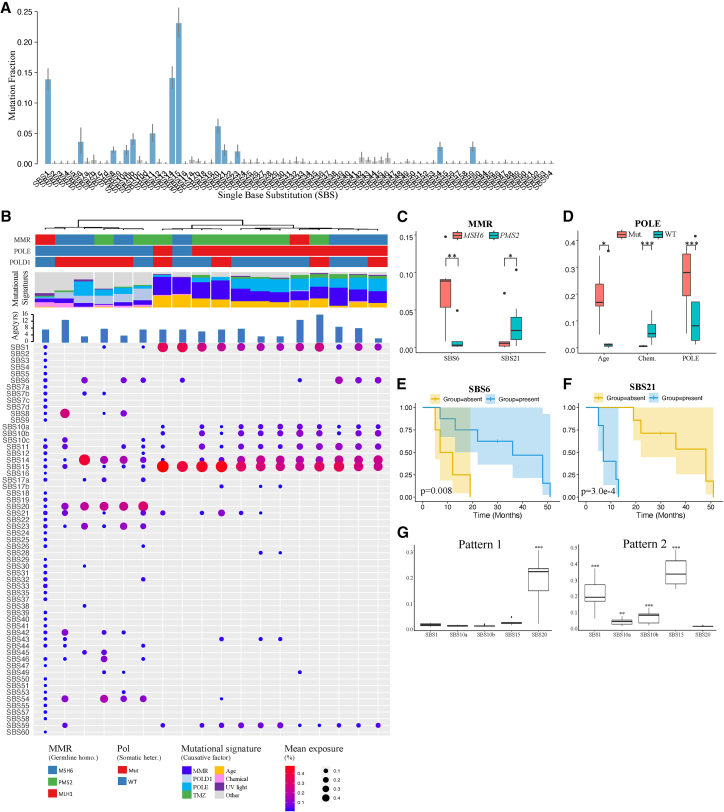
Mutational signatures and clinical outcomes of CMMRD-associated HGGs (A) An overview of single base substitutions (SBS) signatures identified in CMMRD-associated HGGs. Mutational signatures that were present were colored blue. The *x* axis labels (SBS1 to SBS94) correspond to established COSMIC SBS mutational signatures (version 3.2), as defined in the COSMIC database. (B) Hierarchical clustering of CMMRD-associated HGGs based on mutational signatures suggested two clusters representing distinct mutational patterns. Upper, hierarchical clustering dendrogram and mutational profile; middle, mutational signatures; lower, mean exposure of SBS signatures. (C) Comparison of MMR signatures between MSH6 and PMS2 tumors. Only SBS with significant differences between the subgroups were plotted. (D) Comparison of mutational signatures between POLE-wildtype and POLE-mutant tumors. (E) Kaplan-Meier curves of PFS comparing the SBS6 absent and present cases. (F) Kaplan-Meier curves of PFS comparing the SBS21 absent and present cases. (G) Comparison of MMR signatures between two mutational patterns. Only SBS with significant differences between the subgroups were plotted. Continuous variables were compared using the Mann-Whitney U test. Data are presented as boxplots showing the median (center line), interquartile range (box), and outliers (points). ∗*p* < 0.05, ∗∗*p* < 0.01, ∗∗∗*p* < 0.001.

Recent large-scale studies, notably by the ICGC/TCGA Pan-Cancer Analysis of Whole Genomes (PCAWG), have identified a range of mutational associated with endogenous and exogenous mutagenic processes in cancer genomes.^31^ Among these, single base substitution (SBS) signatures, defined as characteristic patterns of point mutations, represent the most common mutation type across cancers. We then investigated the mutational process and patterns of CMMRD-associated HGGs. CMMRD-associated HGGs exhibited significant enrichment of MMR, *POLE*, and *POLD1*-related mutational signatures, accounting for a total of 57.2% ± 14.9% of all mutational signatures identified (Figures 2A, 2B, S4 and S7). The most prevalent signatures included SBS6, SBS10(a/b/c/d), SBS14, SBS15, SBS20, SBS21, SBS26, SBS28, and SBS44, which represented the main mutation processes in CMMRD-associated HGGs. Among these, SBS6, SBS14, SBS15, SBS20, SBS21, SBS26, and SBS44 have been associated with defective MMR and were characterized predominantly by C>T transitions at NpCpG sites. SBS14 is also linked to concurrent defects in MMR and *POLE*, and is primarily composed of C>A transversions in NCT trinucleotide contexts.^32^^,^^33^ The cause of SBS28 is unclear, but it is often seen alongside SBS10a/b, contributing a high mutation burden. In contrast, it is less prominent in samples without SBS10a/b, suggesting a link to POLE exonuclease domain mutations.^34^ Notably, *POLE*-related signatures comprised 20.6% ± 13.7% of total signatures, while *POLD1*-related signatures accounted for only 6.9% ± 11% (*p* = 2.1e-4) (S5). This may suggest a more substantial mutagenic effect from *POLE* variants in these tumors. SBS10a and SBS10b were associated with aberrant POLE activity, characterized predominantly by C>A mutations (SBS10a) and C>T mutations (SBS10b). SBS10c and SBS10d, on the other hand, were linked to defective proofreading due to POLD1 variation and often co-occurs with other MMR deficiency-associated signatures.^35^^,^^36^ Age-related signature SBS1 was also notable, making up 13.9% ± 11.9% of all signatures. SBS1 was described as “clock-like” signature, since the number of mutations attributed to it correlates with the age of cancer diagnosis, reflecting the accumulation of mutations over time. It arises from the spontaneous deamination of 5-methylcytosine to thymine at CpG sites and has been associated with a poor prognosis in certain cancers.^37^^,^^38^ Only a relatively small proportion of mutational process were found related to chemotherapy and chemical exposure.

Table S7. TMB and mutational signatures of the CMMRD-associated HGGs

Importantly, we identified distinct signatures in CMMRD-associated HGGs across different MMR subgroups, indicating potential implications for patient prognosis.

Specifically, *MSH6* HGGs exhibited a higher proportion of SBS6 (7.6% ± 4.4%) compared to *PMS2* HGGs (0.9% ± 1.5%) (*p* = 1.2e-3), with significant associations found between the absence (<1%) of SBS6 and shorter PFS (*p* = 0.008). Conversely, *PMS2* HGGs showed significantly enriched SBS21 compared to *MSH6* HGGs (*p* = 0.042), and its presence was associated with significantly shorter PFS (*p* = 3.0e-4) (Figures 2C–2F). Distinct mutational patterns within *PMS2* subgroup have previously been associated with patient OS. Ercan et al. reported that patients harboring frameshift or truncating variants exhibited poorer OS than those with missense variants.^17^ These findings highlighted the distinct mutational processes among MMR subgroups and their prognostic relevance, warranting the need for further investigation in larger cohorts.

After exploring the mutational signatures among MMR subgroups, we then compared the signatures between *POLE* mutant and wild-type samples (Figure 2D). As expected, we observed significantly enriched *POLE* related signatures (SBS10a/SBS10b) in *POLE* mutant samples (*p* = 6.3e-5). These mutant samples also exhibited a significantly higher proportion of age-associated signatures compared to wild-type samples (19.0% ± 7.9% vs. 5.9% ± 13.3%) (*p* = 0.011). Notably, while signatures associated with chemotherapy and chemical exposure were less prominent overall, the absence of dominant *POLE*-related signatures may contribute to the relative increase these signatures in *POLE* wild-type tumors, which showed a significantly higher proportion of these signatures compared to *POLE* mutant tumors (5.8% ± 4% vs. 0.5% ± 0.2%, *p* = 6.3e-5).

To summarize the mutational patterns of CMMRD-associated HGGs, we performed hierarchical clustering based on the signature fractions. Two distinct clusters of mutational patterns were identified, which were potentially influenced by *POLE*/*POLD1* status (Figure 2B). Mutational pattern 1 showed a significantly higher proportion of *POLD1*-related signature SBS20 (*p* = 1.1e-4), while mutational pattern 2 was characterized by a higher prevalence of *POLE*-related signatures SBS10a (*p* = 1.3e-3) and SBS10b (*p* = 2.2e-4) (Figure 2G). Additionally, mutational pattern 2 showed a high enrichment of SBS15 (33.8% ± 8.5% vs. 1.8% ± 1.1% in Pattern 1, *p* = 1.1e-04), and SBS1 (20.4% ± 9% vs. 0.8% ± 0.6%, *p* = 1.1e-04). No significant differences in the PFS between these two mutational patterns were observed. Combined with our previous findings, these results suggested that *POLE*/*POLD1* may be a key factor affecting the mutational patterns observed in CMMRD-associated HGGs, while some MMR deficiency-related signatures may have prognostic implications.

### Impact of driver mutations on tumor evolution through positive selection

**Figure 3:**
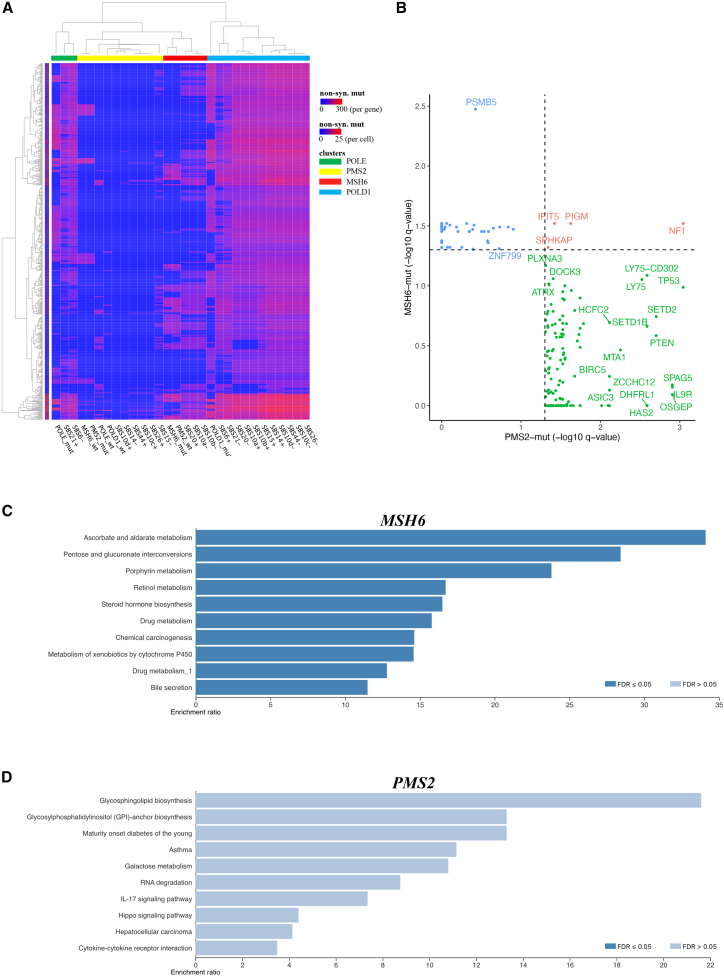
Mutations under positive selection in tumor evolution across molecular subgroups (A) Hierarchical clustering of CMMRD HGG subgroups and positively selected genes. Samples were classified into subgroups based on MMR and POLE/POLD1 mutations or the status of MMR and POLE/POLD1 signatures. Rows: positively selected genes. Columns: molecular or mutational signature subgroups. The color gradients in each cell shows the mutation count for each gene in each subgroup, while the color gradients to the left of the row shows the total mutation count for each gene. Both rows and columns were clustered based on the mutation frequencies. (B) Comparison of positively selected genes between MSH6 and PMS2 tumors. Dushed lines: q = 0.05. Blue dots: positively selected genes in MSH6 tumors exclusively. Green dots: positively selected genes in PMS2 tumors exclusively. Red dots: positively selected genes in both MSH6 and PMS2 tumors. (C) Pathway enrichment analysis of genes under positive selection in MSH6 subgroup. Pathways that were significantly enriched (FDR≤0.05) were highlighted in deep blue. (D) Pathway enrichment analysis of genes under positive selection in PMS2 subgroup.

Tumor evolution depends on genetic variations generated by somatic mutations, which is affected by natural selection. Previous studies have demonstrated that tumor driver genes evolve predominantly by positive selection which increases the accumulation of advantageous mutations for tumor cell survival or proliferation.^39^ To further explore the effect of key driver mutations on “shaping” the somatic mutations of CMMRD-associated HGGs, we estimated the somatic mutations that have been positively selected by tumor evolution for *MSH6*, *PMS2*, *POLE*, and *POLD1* subgroups, respectively (S8). We then performed hierarchical clustering based on the mutation counts in all positively selected genes for each molecular or mutational signature subgroup, and identified 4 distinct clusters (green, yellow, red, and blue (Figure 3A).

Table S8. List of genes detected under significant positive selection in each CMMRD HGG subgroup

Notably, *MSH6*, *PMS2*, *POLE*, and *POLD1* mutant subgroups clustered into these 4 distinct clusters, respectively, suggesting the vast differences in the positive selection among these molecular subgroups (Figure 3A). We further compared the positively selected genes of *MSH6* and *PMS2* subgroups and found only a very small number of shared genes between the two subgroups, including *NF1*, *SPHKAP*, *IFIT5*, *PIGM* (Figure 3B). The majority of positively selected genes were found specific to either *MSH6* or *PMS2* samples. KEGG analysis suggested 10 significantly enriched pathways of positively selected genes in the *MSH6* subgroup (FDR < 0.05), whereas no specific pathways were found enriched in the *PMS2* subgroup (Figures 3C and 3D). These results indicated that MMR and *POLE*/*POLD1* may be crucial in affecting the mutational process and tumor evolution in CMMRD-associated HGGs, leading to different mutational spectrum in tumors.

### Clonal architecture and mutational dynamics in CMMRD-associated HGGs

**Figure 4:**
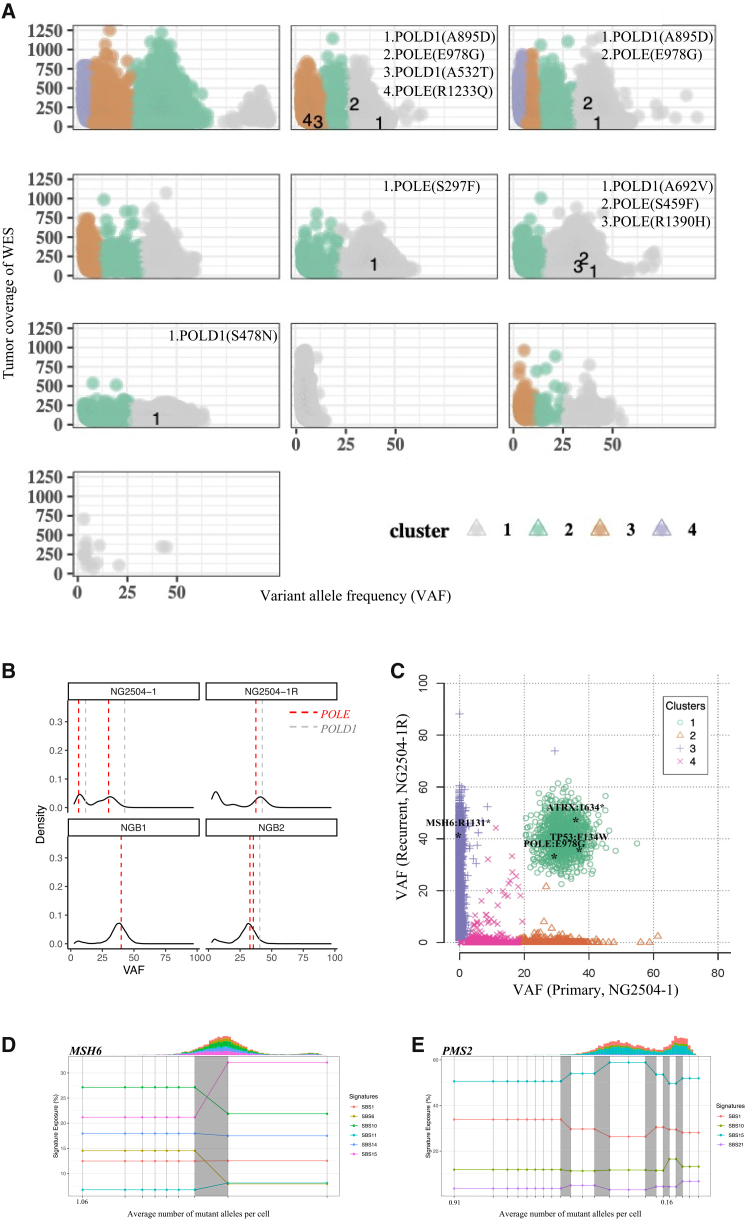
Inferring clonal architecture of CMMRD-associated HGGs (A) Clustering of somatic mutations in CMMRD-associated HGGs based on variant allele frequency (VAF). Each point represents a somatic mutation and were clustered into mutation clusters labeled with different colors, representing distinct subpopulations of tumor cells. (B) Kernel density plots of VAFs indicated the timing of POLE/POLD1 mutations during the mutational process. POLE mutations tend to occur in the VAF center of the mutation clusters, suggesting that they occurred concurrently with the somatic mutation bursts in these ultra-hypermutated cancers. (C) Clustering of somatic mutations for paired primary-recurrent CMMRD-associated HGGs. The primary tumor (NG2504-1) and the recurrent tumor (NG2504-1R) were estimated to have a shared founding clone harboring ATRX, TP53, and POLE mutations. The recurrent tumor had a distinct subclone (purple) harboring an additional MSH6 stop-gained mutation. Representative cases for mutational signature activity trajectories in *MSH6* (D) and *PMS2* (E) subgroup. Darker shades indicate higher density of changepoints.

To infer the clonal architecture of CMMRD-associated HGGs, we conducted mutational clustering based on the variant allele frequencies (VAFs) for each sample. Mutation clusters representing the clonal marker variants were identified, which indicated subclonal populations among the tumor (Figure 4). Detailed information of the clusters was shown in S9. Clonal *POLE*/*POLD1* mutations were observed in all *POLE*/*POLD1* mutant cases, indicating a loss of proofreading function across the tumor cell population (Figure 4A). Additionally, *POLE* mutations showed a VAF around the peak of somatic mutation bursts (Figure 4B). Again, this observation suggested a critical role of *POLE* mutations in shaping the mutational process during tumor evolution.

Table S9. Clonal architecture in CMMRD HGGs

To investigate the clonal architecture of a recurrent CMMRD HGG, we performed mutational clustering for paired primary and recurrent HGGs (Figure 4C and S10). Notably, *POLE* p.E978G, *TP53* p.F134V, *ATRX* p.1634∗ mutations were observed in a shared founding clone, suggesting them as common tumor drivers in both primary and recurrent tumors. Interestingly, an additional stop-gained mutation in *MSH6* (p.R1331X) were identified in the subclone of the recurrent tumor exclusively. Moreover, we observed that deleterious mutations tended to accumulate in specific pathways. Specifically, both the primary and recurrent tumors harbored various mutations in *CIC* (Initial: R440C, R190H, progressed: G365S) and in Ras-PI3K pathway (Initial: *PIK3CA* G106D, *NF1* R156H, progressed: *PIK3CA* C90Y, *NF1* V1772F), while the progressed tumor had additional mutations in Ras-PI3K pathway (*RB1* R445∗, *PTPN11* R502W) and mismatch repair pathway (*MSH6* R1331∗).

Table S10. Clustering analysis of paired primary-recurrent CMMRD-associated HGGs

To gain insights into the mutational dynamics during tumor evolution, we analyzed changes in MMR/*POLE*/*POLD1* mutational signatures over time within the *MSH6* and *PMS2* subgroups (Figures 4D, 4E, and S6). Our observations revealed that the proportion of signatures varied significantly throughout tumor evolution, particularly during periods of somatic mutation bursts. As expected, SBS6 was exclusively observed in *MSH6* whereas SBS21 was predominantly detected in *PMS2* subgroups. Both of these PFS-related mutational signatures, although relatively weak, displayed a persistent presence throughout the evolutionary trajectory. These findings further underscored the distinct mutational process in tumor evolution *MSH6* versus *PMS2* CMMRD-associated HGGs.

### Clonal evolution models revealed distinct patterns of evolution in *MSH6*/*PMS2* subgroup

**Figure 5:**
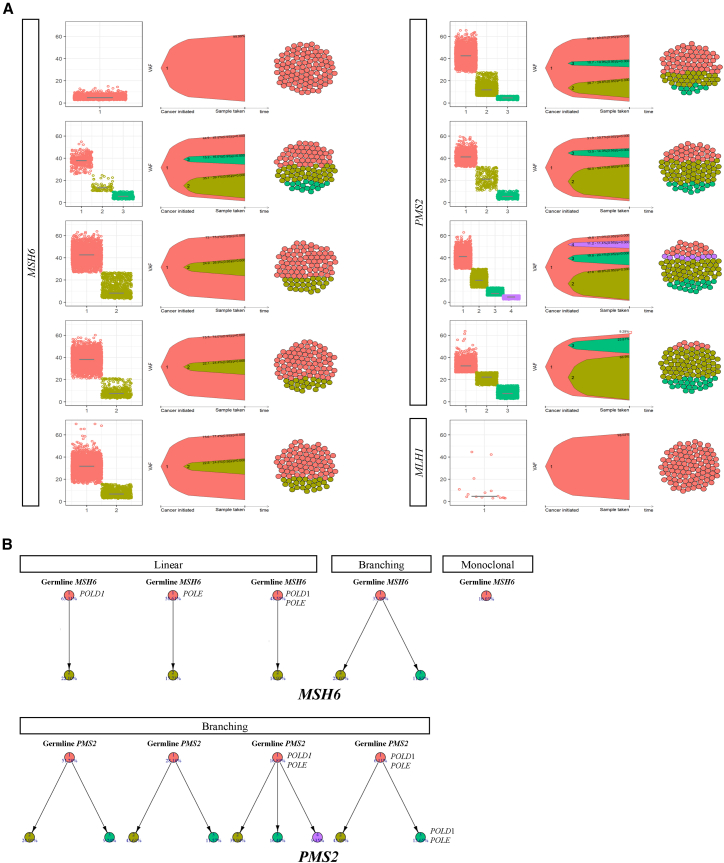
Clonal evolution in CMMRD-associated HGGs (A) Reconstruction of clonal evolution model in CMMRD-associated HGGs across MMR subgroups based on inferred clonal architecture. Left: Mutation clusters; middle: fish plot showing the clonal dynamics during the tumor development; right: sphere of cells indicating the clonal subpopulations of the sequenced sample. (B) Phylogenetic trees representing the evolutionary trajectories of CMMRD-associated HGGs in MSH6 and PMS2 subgroup.

Using the results of estimated clonal architecture, we further reconstructed the clonal evolution models of CMMRD-associated HGGs. Patterns of evolutionary trajectories and composition of tumor cell population at the time of sample taken were shown for *MSH6*, *PMS2*, and *MLH1* subgroup in Figure 5A. We found that *MSH6* and *PMS2* tumors had distinct patterns of clonal evolution. *PMS2* mutant CMMRD-associated HGGs demonstrated characteristics of branching evolution, in which multiple subclones evolved in parallel, whereas only 1 *MSH6* mutant case showed a similar pattern (*p* = 0.048) (Figure 5B). In contrast, a pattern of linear evolution was observed in *MSH6* cases only.

The reconstructed model also revealed the evolutionary history of key mutations, and we annotated germline MMR mutation and somatic *POLE*/*POLD1* mutations in the phylogenetic trees in Figure 5B. Notably, *POLE*/*POLD1* mutations tended to occur in the founding clones, suggesting their early occurrence during the mutational process. Additionally, when inspecting previously reported HGG drivers, subclonal driver mutations were observed in *PMS2* CMMRD-associated HGGs exclusively, while HGG drivers were found clonal in other subgroups (S11). These observations were consistent with the branching evolution signature of *PMS2* tumors, as one of the hallmarks of branching evolution is the continued accumulation of subclonal driver mutations. Furthermore, characteristics of convergent evolution, in which different clones mutate the same driver gene, were observed in *NF1*, *ATRX*, *IDH1/2*, *NES*, *PDGFRA*, *PTEN*, and PI3K/Akt/mTOR pathway.

Table S11. Clonal and subclonal driver mutations

Allele-specific copy number (ASCN) analysis was performed to estimate the cellular fraction of copy number alterations in CMMRD-associated HGGs (S1 and S4). As expected, clonal copy number calls corresponding to a normal diploid state (total CN = 2, minor CN = 1) were observed in all samples. Notably, all CNVs in CMMRD HGGs were estimated to be subclonal (mean cellular fraction, 0.33 ± 0.18), with the exception of a single focal loss on chromosome X (chrX: 284,212–2,726,273) in the *MLH1* tumor (NG11078), which was estimated to be clonal.

### Survival of founding clone during tumor recurrence

**Figure 6:**
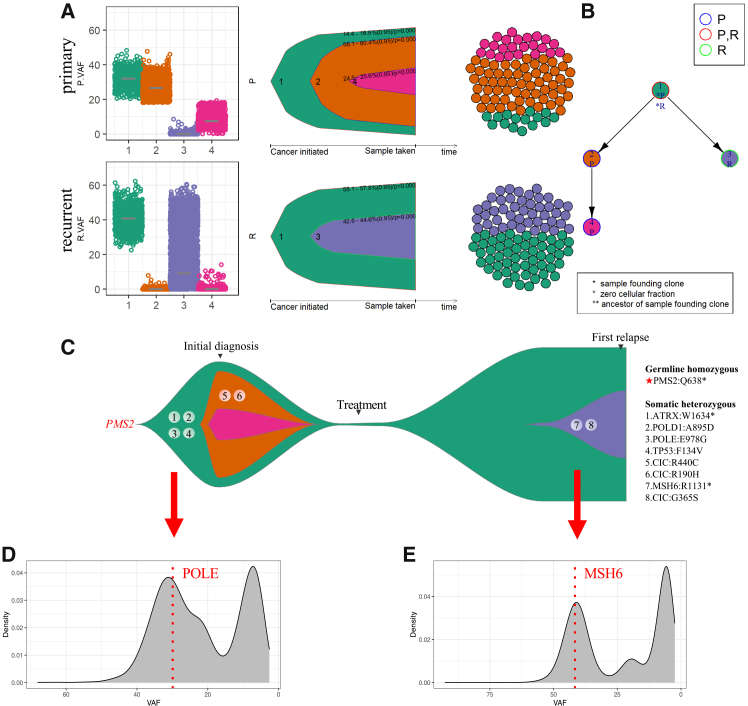
Evolutionary trajectory in CMMRD-associated HGG recurrence (A) Reconstruction of clonal evolution model in paired primary-recurrent tumors. Left: cluster analysis of somatic mutations; middle: Fish plot showing clonal dynamics; Right: sphere of cells representing clonal subpopulations. (B) Phylogenetic trees representing the evolutionary trajectories of the primary-recurrent tumor pair. P, primary tumor. R, recurrent tumor. (C) Representative of the evolutionary trajectory in CMMRD-associated HGG from tumor occurrence to recurrence. The founding clone was found to survive conventional treatment whereas all subclones (orange, purple) in the primary tumor were eliminated. The recurrent tumor shared the same clonal origin and evolved a distinct subclone harboring MSH6 R1131∗ and CIC G365S mutations. Treatment: radiotherapy with temozolomide and chemotherapy with ciplatin and etoposide. Timing of the somatic (D) POLE and (E) MSH6 mutations occurred around the time of mutation bursts in the primary and recurrent tumors, respectively.

To investigate the evolutionary trajectory of tumor recurrence in CMMRD HGG patients, we reconstructed clonal evolution model based on paired primary-recurrent tumors (Figures 6A and 6B). The primary tumor originated from a founding clone harboring a germline *PMS2* mutation and somatic *POLE*, *POLD1*, *ARTX*, and *TP53* mutations, which were predicted deleterious and were inferred as the early events of the tumorigenesis, indicating their important role in driving the CMMRD HGG (Figure 6C). An enrichment of deleterious *TP53* and *ATRX* mutations in CNS cancers compared to other cancers in patients with CMMRD, has been previously reported.^17^ Notably, the first somatic mutations burst occurred around the acquisition of the somatic *POLE* mutation, indicating the timing of hypermutation formation in CMMRD-associated HGGs (Figure 5D). The founding clone subsequently evolved into subclones and accumulated additional mutations, particularly in the tumor suppressor gene *CIC* (R440C, R190H), which has been related to the tumorigenesis of GBM through RTK/Ras/ERK signaling. Consistent with this, an enrichment of deleterious mutations in the RAS-MAPK pathway among CNS tumors in CMMRD patients has been observed previously.^17^ Importantly, although all subclones in the primary tumor were eliminated after radiotherapy and chemotherapy, the founding clone survived and continued to evolve, which resulted in tumor recurrence eventually (Figure 6C). Of note, it was not until the occurrence of the new subclone, that the recurrent tumor had its first somatic mutation burst, which resulted in even higher TMB than the already ultra-hypermutated primary tumor. Coincidentally, the timing of this somatic mutation burst was also around the time when the additional *MSH6* mutation occurred (Figure 6D), which may suggest the potential role of this somatic *MSH6* mutation as a “mutator mutations”.^40^

## Discussion

In this study, we identified mutational signatures representing the major mutational process in CMMRD-associated HGGs and two mutational patterns determined by *POLE*/*POLD1* status. We also observed distinct signatures of *MSH6* and *PMS2* subgroups which were associated prognosis, and distinct clusters of positively selected genes among molecular subgroups. Through reconstruction of clonal architecture, we investigated the distribution of key mutations, and established *POLE* and *POLD1* as clonal events. Furthermore, we demonstrated the evolutionary history of both primary and recurrent CMMRD-associated HGGs. Primary tumors were found to be driven by germline MMR mutations, and potentially a subset of mutations in *NF1*, *ATRX*, *IDH1/2*, *NES*, *PDGFRA*, *PTEN*, and PI3K/Akt/mTOR pathway. The recurrent tumor, although found to be originated from the same founding clone, evolved subclones with additional *MSH6* mutation and somatic mutation bursts. *PMS2* and *MSH6* subgroup showed significant differences in evolutionary patterns, and *PMS2* tumors were also found to carry subclonal driver mutations. We then tracked changes in mutational signatures during tumor evolution, and noticed a persistent signal of PFS-associated signatures SBS6/SBS21. By providing comprehensive insights into the mutational signature and clonal evolution of CMMRD-associated HGGs, this study may help support and advance ongoing efforts in the field of these rare malignancies.

Few studies have focused on the molecular profile of CMMRD-associated HGGs, and the mutational signature of these tumors have not been well characterized. Guerrini-Rousseau et al. reported poor prognosis in a cohort of 49 CMMRD patients, who had a variety of brain cancers including HGG and embryonal tumors, and proposed clinicopathological factors that worth noting for diagnosis.^5^ Indraccolo et al. observed partial or total *MSH6* expression decrease in 12 recurrent GBMs with distinct molecular profiles such as high TMB, telomere shortening, *MGMT* methylation, and highly heterogeneous MHC class I expression in these cases.^41^ A more recent study of Suwala et al. analyzed the genomic profile and methylation signature of 32 IDH-mutant gliomas in which the majority have been diagnosed with Lynch/CMMRD.^24^ Although only 7 cases were confirmed as CMMRD-associated HGGs, their work revealed that primary mismatch repair deficient IDH-mutant astrocytoma should be characterized as a distinct subtype with poor clinical outcome. They also observed pathogenic mutations in *TP53*, *RB1*, and in RAS/PI3K/AKT pathway, which was concordant with our results that 77.8% CMMRD-associated HGGs harbored *TP53* mutation while at least 40% cases harbored synonymous mutation in *TP53*, *NF1*, *PIK3CA*, *PTEN*, *PDGFRA*, *RB1*. It is worth noting that we observed much higher frequencies of *NF1* (77.8%, 14/18) and *PTEN* (50%, 9/18) mutations. Moreover, we assessed the genomic profile and mutational signature of *PMS2* subgroup, whereas no *PMS2* cases were included in the previous study of Suwala et al.

Recently, several large studies have investigated the genomic and epigenomic features of these rare tumors.^15^^,^^17^^,^^21^^,^^24^ The study by Negm et al. is the first report of the prevalence of primary mismatch repair deficiency (MMRD) across glioma subtypes in children, adolescents, and young adults using a population-based cohort of 1389 gliomas.^15^ They found that primary MMRD is more common than previously reported in gliomas in these age groups. Notably, they observed particularly poor survival for IDH-mut primary MMRD astrocytomas. Such case can also be observed in our cohort. Early detection and implementation of immunotherapy may improve the outcome for these patients.

Another important large-scale study by Ercan et al. investigated the clinical and biological landscape of CMMRD patients with CNS tumors, including 115 glioblastomas and 25 anaplastic astrocytomas.^17^ They reported distinct prognostic and mutational patterns among MMR subgroups. Notably, they also observed mutational patterns associated with poorer OS in the *PMS2* subgroups. Consistent with their findings, we observed a significant enrichment of SBS21 in the *PMS2* subgroup compared to the *MSH6* subgroup, which was associated with significantly shorter PFS in our cohort. Additionally, Ercan et al. found that patients harboring frameshift or truncating variants had poorer overall survival than those with missense variants, with the most pronounced differences observed in patients carrying germline *PMS2* mutations. Collectively, these findings further support the clinical relevance of distinct mutational signatures within the *PMS2* subgroup. While Ercan et al. compared the genomic and clinical features between *MLH1*/*MSH2* and *PMS2*/*MSH6* subgroup, our study compared the mutational profile and clinical outcomes between the *PMS2* and *MSH6* subgroups. They reported worse OS in *MLH1* and *MSH2* subgroup and predicted OS at age 15 years for patients with PMS2 and *MSH6* mutations. It is worth noting that the age distribution in their cohort differs from ours, encompassing a broader age range—including children, adolescents, and adults—with a median age at diagnosis of 9.8 years. In contrast, our study focused exclusively on pediatric patients, with a younger median age at diagnosis of 5.8 years.

In the present study, we attempted to classify MMR subgroups using hierarchical clustering based on previously established mutational signatures, revealing two subgroups with distinct mutational profiles. Although these mutational subgroups were not associated with patient prognosis in our cohort, hierarchical clustering may hold promise for subgroup classification in studies with larger sample sizes. This approach has been discussed in the study of Suwala et al., in which a distinct group of primary MMRD IDH-mutant astrocytomas was identified through clustering analysis based on DNA methylation profiles, despite previous failure in studies with smaller sample size.^24^ Their analysis demonstrated that clustering analysis could clearly distinguish primary MMR subgroup from other gliomas, including acquired MMR-deficient tumors, suggesting distinct mechanism underlying oncogenesis. Thus, hierarchical clustering might be a promising strategy for classifying MMR subgroups. Its clinical applicability requires further investigation, particularly in larger cohorts.

To date, studies related to the clonal structure or tumor evolution of CMMRD HGG tumors are sparse. Dodgshun et al. expanded a cohort from the international replication repair deficiency consortium, and investigated the temporal and spatial intratumoral heterogeneity in HGGs in the context of several RRD syndromes including CMMRD, Lynch, and POLE.^42^ However, this study mainly focused on the hypomethylation patterns instead of clonal evolution. The former was considered relatively stable during the tumor evolution with little intratumoral heterogeneity, whereas our observations on the dynamic of mutational signature indicated noticeable changes particularly during the period of somatic mutation bursts. Interestingly, they observed acquired driver mutations, in particularly *POLE* mutation, in the relapsed tumor that was capable of altering the methylation profile of RRD tumors. We had a similar observation but in the perspective of mutational process, in which somatic mutation bursts tend to occur around the time of *POLE* and *MSH6* acquisition.

Previous studies have reported TMZ induced MMR-deficiency in HGGs, and acquired MMR deficiency was suggested as an attributor of TMZ-resistance closely related to the tumor recurrence.^25^^,^^43^^,^^44^^,^^45^^,^^46^ For example, Cahill et al. analyzed 54 GBM cases and observed significantly increased *MSH6* mutations in recurrent cases, whereas no *MSH6* mutation was observed in the pretreated tumor.^46^ McFaline-Figueroa et al. reported the role of *MSH2* in temozolomide (TMZ) resistance in recurrent GBM.^47^ Intriguingly, we also identified an acquired *MSH6* stop-gained mutation in the recurrent CMMRD HGG. It is plausible to assume that this *MSH6* mutation may have resulted in acquired MMR deficiency if a constitutional MMR deficiency had not already occurred. MMR can be a key mediator for alkylating agents response. In the absence of MGMT-mediated repair and intact MMR, TMZ exposure can cause the emergence of hypermutation via a burst of transitions in the context of DNA replication and may even promote tumor progression. A recent large-scale cohort study by Touat et al. suggested that MMR deficiency can drive hypermutation and chemotherapy resistance in gliomas through tumor evolution based on mutational signature and clonal evolution analysis.^19^ They concluded that hypermutated gliomas with acquired MMR-deficiency tend to be subclonal. Such clonal structure can lead to a compromised response to immunotherapy, as demonstrated in their retrospective analysis. Acquired MMR defects and hypermutation phenotype have been observed during the treatment of low-grade gliomas treated with TMZ as well.^48^ These studies highlighted the urgent need to study the clonal structure and evolution of CMMRD-associated HGGs, which may aid in optimizing therapeutic strategies of the patients.

*POLE*/*POLD1* mutations are known to trigger hypermutations in CMMRD malignancies, however, the underlying mutational process remains elusive. Shlien at al. performed clonality analysis of 6 ultra-hypermutated CMMRD tumors including 5 HGGs and investigated the timing of *POLE*/*POLD1* mutations with respect of other somatic mutations in the genome.^27^ Based on the observation of VAFs, they suggested that *POLE*/*POLD1* driver mutations occurred in the earliest possible clone. This observation was confirmed by our reconstructed clonal evolution model, which mathematically estimated the occurrence of key driver mutations along the phylogenetic tree, and indicated an early occurrence of *POLE*/*POLD1* mutations in all *POLE*/*POLD1* mutant cases. Moreover, we explored the impact of these mutations on both the molecular and clinical profile of CMMRD-associated HGGs, and found that *POLE*/*POLD1* mutations tended to affect the mutation patterns rather than patient prognosis. Notably, the clonal evolution pattern may differ between primary and recurrent CMMRD-associated HGGs. Our recurrent tumor evolution model suggests that, although recurrent tumors also carried POLE/POLD1 mutations, these mutations likely occurred in the founding clone of the primary tumor. Furthermore, unlike the coincidence of mutation burst with POLE/POLD1 mutations observed in the primary tumors, the mutation burst in the recurrent tumor was estimated to occur around the emergence of the subclonal MSH6 mutation. These findings underscore the need for further investigation into the distinct evolutionary patterns of recurrent CMMRD HGGs. The clinical implications for these evolutionary dynamics on therapeutic strategies remain to be elucidated. Additionally, several rare mutations in *POL*E and *POLD1* were observed in our cohort. Most of these mutations are located within the polymerase domain of *POLE* (p.S964Y, p.R680C, and p.R847W) and *POLD1* (p. A692V and p.A915V) and were predicted deleterious. Experimental validation of their functional impact is urgently needed. Laboratory approaches, such as the development of conditional knockout models, may aid in confirming the pathogenicity and biological significance of these mutations.

The coexistence of somatic *POLE*/*POLD1* mutations alongside germline MMR mutations in CMMRD patients represents a convergence of proofreading deficiency and mismatch repair failure, driving profound genomic instability with potential clinical implications.^27^^,^^49^ *POLD1* and *POLE* encode subunits of the polymerase δ and ε, respectively. Mutations within their exonuclease domains, especially at conserved residues (e.g., *POLE* p.P286R, p.V411L), impair the proofreading function of the polymerase, leading to base substitution accumulation and elevated mutation burden.^50^ Meanwhile, germline MMR (*MSH6*, *PMS2 MLH1*, and *MSH2*) mutations disrupt the post-replicative correction of mismatched base pairs and small indels. MMR and *POLE*/*POLD1* are functionally linked through the MutSα-MutLα-dependent repair mechanism, wherein MutSα (*MSH2*/*MSH6*) recognizes mismatches and recruits MutLα (*MLH1*/*PMS2*) to remove replication error, followed by resynthesis by Pol δ or ε.^51^^,^^52^^,^^53^^,^^54^^,^^55^ Notably, the combined loss of these functions generates distinct signatures, reflecting a unique interaction between these two repair systems.^33^ Together, the concurrent loss of mismatch repair function and polymerase proofreading capacity leads to a hypermutator or even ultramutator phenotype, with distinct biological and therapeutic relevance.^25^^,^^56^^,^^57^

In conclusion, our study provides comprehensive insights into the mutational signature and clonal evolution of CMMRD-associated HGGs. Our results revealed distinct mutational patterns and evolutionary trajectories underlying different CMMRD HGG molecular subgroups associated with clinical outcomes. This study has the potential to aid the clinical management of the patients and shed light on the tumorigenesis and progression of HGGs in CMMRD.

## Resource availability

### Lead contact

For further information and requests for resources and reagents, please contact Ahmet Okay Çağlayan (ahmetokay.caglayan@deu.edu.tr).

### Materials availability

This study did not generate new unique reagents.

### Data and code availability

• DNA sequencing data generated in this study have been deposited at European Genome-phenome Archive (EGA), under the accession number: EGAS50000001506 and are publicly available through a DAC-managed access policy with no additional restrictions. Due to local regulatory requirements, raw sequencing data generated in this study are not publicly released. Summarized, non-identifiable somatic variant data are provided in Table S14 and are openly accessible.
  Table S14. Summary of somatic variants identified by WES in CMMRD-associated HGGs
• Data and any additional information required to reanalyze the data reported in this paper is available from the lead contact upon request.
• Any data request will be made through the lead contact. The study did not generate any original code.

### Limitations of the study

This study may be subject to several limitations. First, we only included 25 CMMRD-associated HGGs and among these only 18 underwent WES. The limited sample size may thus reduce the reliability of the analysis and the applicability of the findings. However, this was mainly due to the rarity of these tumors. In fact, previous publications on CMMRD-associated HGGs were mostly case reports, whereas the sample sizes of cohort studies were relatively small.^27^^,^^42^ Thus, our findings are expected to be of great value for improving the understanding of this disease given a comparable sample size. In addition, the analysis of the mutational process and evolutionary trajectories of CMMRD HGG needs to be validated in subsequent studies, and the underlying mechanism and clinical significance is worth further investigation. To our knowledge, this is the first study to perform clonality analysis of CNVs in CMMRD-associated HGGs. However, the influence of CNVs on evolutionary trajectories requires further validation.

## Acknowledgments

This work was supported by Gregory M. Kiez and Mehmet Kutman Foundation. C.L. was supported by the 10.13039/501100021171Guangdong Basic and Applied Basic Research Foundation (2021A1515111051). The authors thank the reported family for participating in this study.

## Author contributions

A.O.C. and M.G. designed the study. A.O.C. and K.B. conducted whole-exome sequencing. A.O.C. and C.L. performed exome data analysis. A.O.C. and C.L. performed and Sanger sequencing. A.O.C., Y.K., E.U., B.K., Y.P., N.A.D., A.K., H.P., and J.R.O. performed sample and patient data procurement. A.O.C., Y.K., E.U., B.K., Y.P., N.A.D., A.K., H.P., and J.R.O. collected and investigated clinical data. C.L. and A.O.C. wrote manuscript. A.O.C. and M.G. revised the manuscript. All authors read and approved the final manuscript.

## Declaration of interests

The authors declare no competing interests.

## STAR★Methods

### Key resources table

**Table:** 

REAGENT or RESOURCE	SOURCE	IDENTIFIER
**Deposited data**		

DNA sequencing data of CMMRD-associated HGGs	This paper	EGA: EGAS50000001506
DNA sequencing data of CMMRD-associated HGGs	Suwala et al.^24^	Supplement: https://link.springer.com/article/10.1007/s00401-020-02243-6
DNA sequencing data of CMMRD-associated HGGs	Shlien et al.^27^	EGA: EGAD00001000369, EGAS00001001112.

**Software and algorithms**		

Burrows–Wheeler Aligner	Li et al.^58^	v0.5.9-r16
Genome Analysis Toolkit	DePristo et al.^59^	v2.5
The Ensembl Variant Effect Predictor	McLaren et al.^60^	v2.7
COSMIC mutational signature	Alexandrov et al.^61^	v3.2
Sigfit	Gori et al.^62^	v2.2.0
dNdScv	Martincorena et al.^63^	v0.0.0.9
SciClone	Miller et al.^64^	v1.1.1
ClonEvol	Dang et al.^65^	v0.99.11
TrackSig	Rubanova et al.^66^	v0.2.0
FACETS	Shen et al.^67^	v0.6.2
R	https://www.r-project.org	v4.3.3

### Experimental model and study participant details

#### Ethics approval and consent to participate

This study has been reviewed and approved by the institutional review board of Dokuz Eylul University (#2023/31-27). Institutional review board approval for genetic and MRI studies, along with written consent from all study subjects, were obtained from the referring physicians at the participating institutions.

#### Clinical materials and inclusion criteria

Patients with CMMRD and at least one high-grade glioma were included. The diagnosis of each patient was reviewed according to the latest diagnostic criteria for CMMRD recommended by the international consensus working group.^13^ We collected the tumor and blood samples of 11 CMMRD-associated HGG patients and blood samples of 8 family members from 6 CMMRD families. Part of the clinical and radiological findings of these newly collected cases can be found in our previous publication.^1^ We also included 14 cases of CMMRD-associated HGGs from previous studies.^24^^,^^27^ Thus, a total of 25 CMMRD-associated HGGs were included for the analysis. Patient inclusion was not influenced by sex or gender. No influence of sex or gender was observed on the results of the study. Race, ancestry, and ethnicity information were not collected for the participants in this study. Clinical and demographic patient information is provided in .

### Method details

#### Selective tissue dissection and DNA extraction

On H&E-stained sections from FFPE tissue blocks, areas of interest were identified and microscopically dissected to ensure that each sample consisted of more than 70% tumor cells; irrelevant regions such as inflammatory and necrotic areas were excluded. DNA was then prepared using the Allprep DNA/RNA/protein Mini Kit (Qiagen Science, MD). Blood samples were collected from both the patients and the patients’ parents. DNA was extracted from the blood samples using the commercially available Gentra Puregene Blood Kit from Qiagen.

#### Whole-exome sequencing

We performed whole-exome sequencing (WES) of 10 CMMRD-associated HGGs with matched blood samples. NimbleGen 2.1M human exome array (Roche Nimblegen, Inc.) was used to capture the exomes of all samples according to the manufacturer’s protocol with modifications. The data were analyzed as described before.^23^ Briefly, alignment of the reference sequence (version GRCh37) was performed using Burrows–Wheeler Aligner (v0.5.9-r16) and Stampy (v1.0.16).^58^ Mutations were identified using the Haplotyper caller in the Genome Analysis Toolkit (GATK v2.5).^59^ Called variants was then annotated using Ensembl database (v69) and Variant Effect Predictor v2.7 tool.^60^ Genetic variant pathogenicity prediction was performed using SIFT and PolyPhen2.^61^^,^^68^ Called variants were filtered based on reference population databases e.g., National Heart, Lung, and Blood Institute’s Exome Variant Server and 1000 Genomes.^69^ Copy Number Variation (CNV) were assessed using the ExomeCNV tool.^70^ CNV burden was calculated as the number of megabases affected by CNV. TMB was calculated as the total number of somatic mutations per megabase of the targeted region.

Sanger confirmation of germline mutations in MMR genes (*MSH6*, *PMS2*, *MLH1*) in the family members were performed when available (S1). The pathogenicity of variants in *POLE* and *POLD1* were further determined based on previous publications, variant database ClinVar, population databases gnomAD and ExAC.^71^^,^^72^^,^^73^ Passenger variants or variant with undetermined significance in *POLE* and *POLD1* were excluded from the analysis. Mean coverage of 184.1(±36.1) for tumor and 91.3(±25.3) for matching blood, and average percentages of reads at 30× coverage of 94.0%(±2.5%) for tumor and 87.6%(±8.8%) were achieved. The average tumor purity of the sequenced sample was 41.4%(±21.9%).

#### COSMIC mutational signature analysis

Somatic mutations are known as the consequence of multiple mutational processes including defective DNA repair, and to estimate the mutational processes in HGGs with CMMRD, we calculated the fraction of COSMIC mutational signature in each tumor sample (v3.2).^31^ Signature fitting was performed using the Sigfit algorithm (v2.2.0) with default settings which estimates the exposure of samples to predefined mutational signatures based on Bayesian model.^62^ Signatures had an estimated exposure value of “sufficiently non-zero” were considered present. The hierarchical cluster analysis of the mutational signature was performed using the built in hclust function in R version 4.3.3.

#### Positive selection analysis

Positive selection in the somatic evolution in CMMRD-associated HGGs was quantified using dNdScv R package with default settings.^63^ Briefly, the analysis was performed based on the dN/dS method, which measures the ratio of non-synonymous (dN) to synonymous substitutions (dS) representing the strength of natural selection on protin-coding genes.^74^ Genes with q < 0.05 were selected. Genes under positive selection were identified for each molecular subgroup, including *MSH6*, *PMS2*, *POLE*, and *POLD1*, respectively. Furthermore, cases were classified into subgroups based on the presence of MMR/Pol signatures, in which positive selection analysis were performed. The single *MLH1* case was excluded from the positive selection analysis. Positively selected genes were then clustered based on the mutational frequencies across CMMRD HGG subgroups.

#### Clustering of somatic mutations

Tumor subclones can be identified by clustering heterozygous somatic variants based on their cellular prevalence estimated via WES. To identify the number and genetic composition of subclones of HGGs in CMMRD, mutational clustering of somatic mutations in each sample was performed using the SciClone algorithm with a default setting.^64^ Briefly, the variant allele frequency (VAF) for somatic mutations were calculated based on WES. Since the VAF of somatic mutations in CNV-affected regions may not accurately reflect the cancer cell fractions (CCFs), mutations affected by CNV or loss of heterozygosity (LOH) were excluded from the clustering analysis. Mutational clusters representing tumor subclones harboring distinct mutations were then identified by clustering the VAFs using a variational Bayesian mixture model with consideration of CNV/LOH and sequencing read depth. The clonal structure of the paired tumor/relapse pair was analyzed in two-dimensional analysis mode.

#### Reconstruction of clonal evolution

Based on the identified subclones via mutational clustering, we performed clonal ordering and phylogeny tree construction for the HGGs underwent WES using ClonEvol (ver 0.99.11).^65^ The confidence interval (CI) of the VAF of each clone and *p*-value for probabilistic evaluation of clonal ordering with biological constraints were calculated. A phylogeny model with *p* < 0.05 for all subclones was selected. The reconstructed clonal structure and evolution model were visualized using ClonEvol and Fishplot.^75^ To understand how mutation processes change during the tumor evolution, the evolutionary trajectories of mutational signature activity in each CMMRD HGG was reconstructed using TrackSig based on the WES data.^66^ Only validated signatures with a known causative factor that were detected in the tumor were included for the analysis.

#### Allele-specific copy number (ASCN) analysis

ASCN was performed using FACETS.^67^ WES BAM files were used as input and processed with default settings according to the publisher’s instructions. Copy number estimates were filtered by integrating results from the ExomeCNV tool. CNV events with an estimated cellular fraction of 1 were classified as clonal, whereas those with a fraction <1 were classified as subclonal.

### Quantification and statistical analysis

Survival analyses were performed using R package “survminer”. Nominal variables were compared using the Fisher’s exact test (two-tailed). Continuous variables were compared using the Mann-Whitney U test. The *p* value of a multiple comparison was corrected using Bonferroni correction or false discovery rate (FDR) when applicable. A p or q value <0.05 was considered statistically significant. Statistical significance was indicated as follows: ∗*p* < 0.05, ∗∗*p* < 0.01, ∗∗∗*p* < 0.001. Statistical tests were performed using R software (v4.3.3).

## Supplemental information

Document S1. Figures S1–S6

Table S12. WES coverage of the cohort

Table S13. Tumor purity of the WES cohort
