# Supplementary material for: Distinct mutational signature and clonal evolution in constitutional mismatch repair deficiency-associated high-grade gliomas
Source: iScience. 2026 Feb 14;29(3):115029. doi: 10.1016/j.isci.2026.115029 (PMC12964222; doi:10.1016/j.isci.2026.115029)
Supplement: Document S1. Figures S1–S6 [file mmc1.pdf]

iScience, Volume *29*

Supplemental information

Distinct mutational signature and clonal evolution in constitutional mismatch repair deficiency-associated high-grade gliomas

Chang Li, E. Zeynep Erson-Omay, Yavuz Koksal, Ekrem Unal, Buket Kara, Kaya Bilguvar, Yahya Paksoy, Nimetullah Alper Durmus, Ali Kurtsoy, Huseyin Per, John Rosendahl Østergaard, Murat Günel, and Ahmet Okay Çağlayan

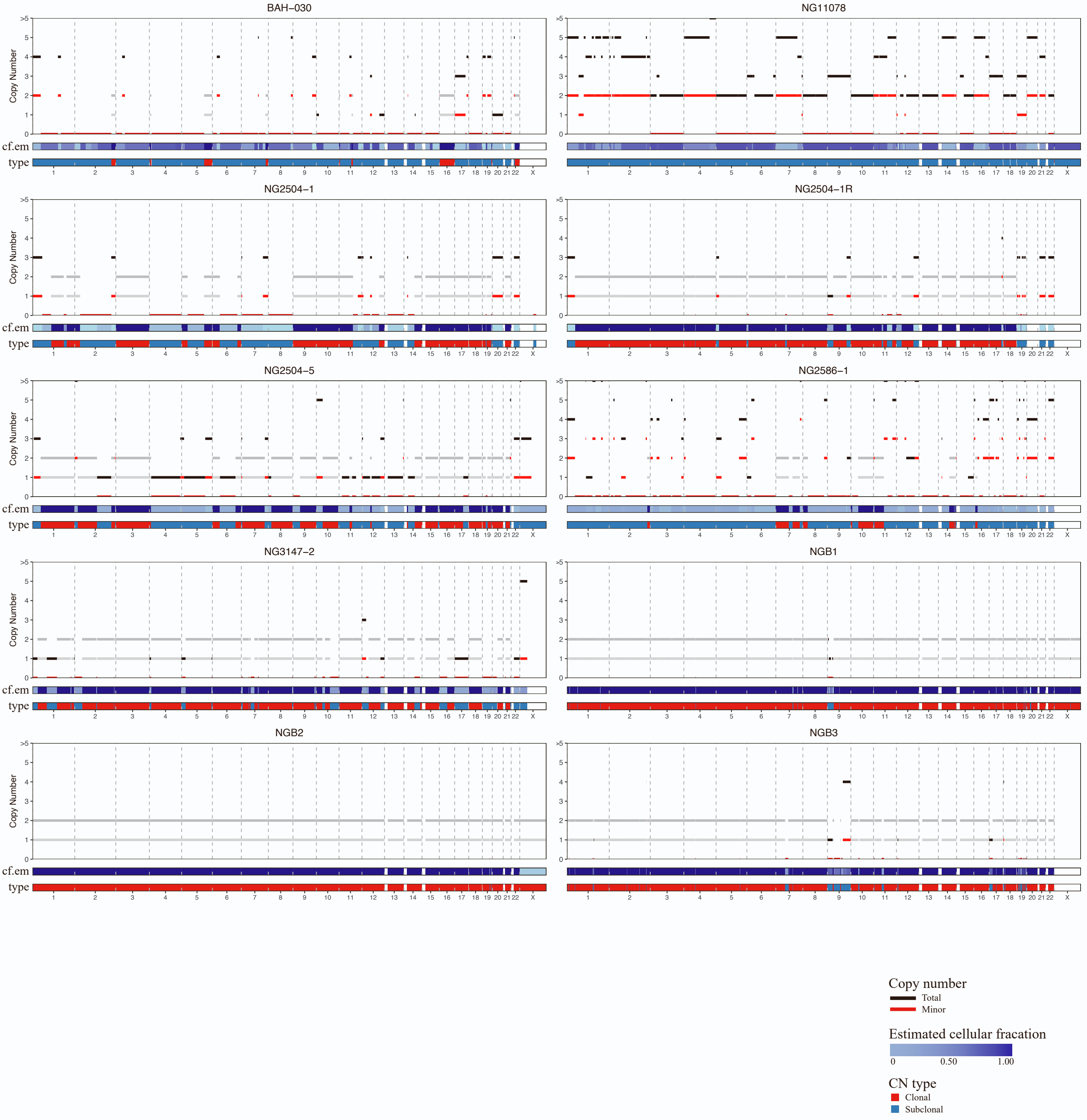

Supplementary. Fig. 1. CNV analysis of CMMRD HGGs underwent WES, Related to Figure 1. Toal and minor copy number for each sample is plotted at the top. The estimated cellular fraction (cf.em) profile is plotted at the bottom, revealing clonal (cf.em=1) and subclonal (cf.em<1) copy number events.

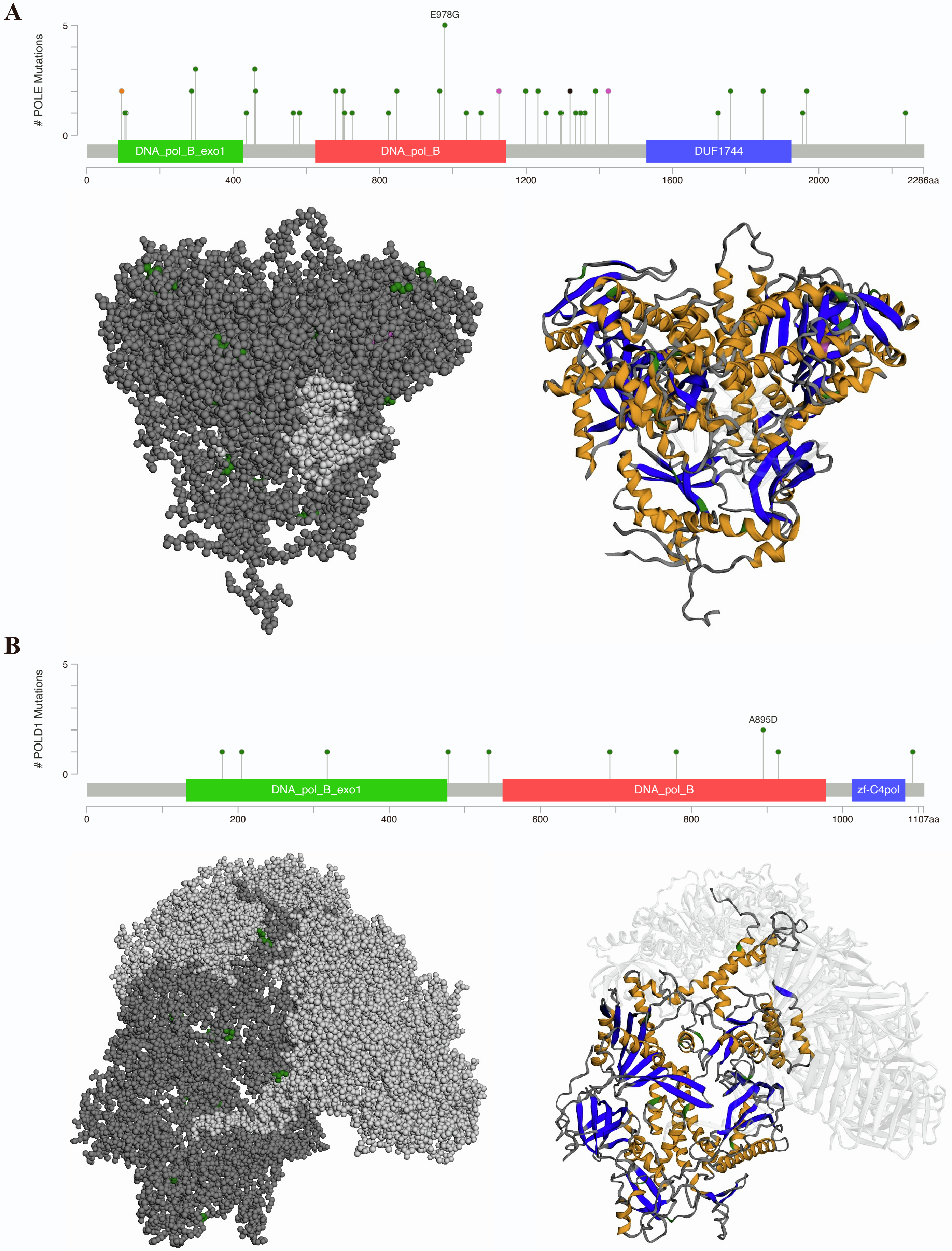

Supplementary. Fig. 2. Non-synonymous mutations in *POLE*/*POLD1*, Related to Figure 1. Non-synonymous mutations in (A) *POLE* and (B) *POLD1* and their predicted effects in the protein structure in 18 CMMRD HGGs underwent WES.

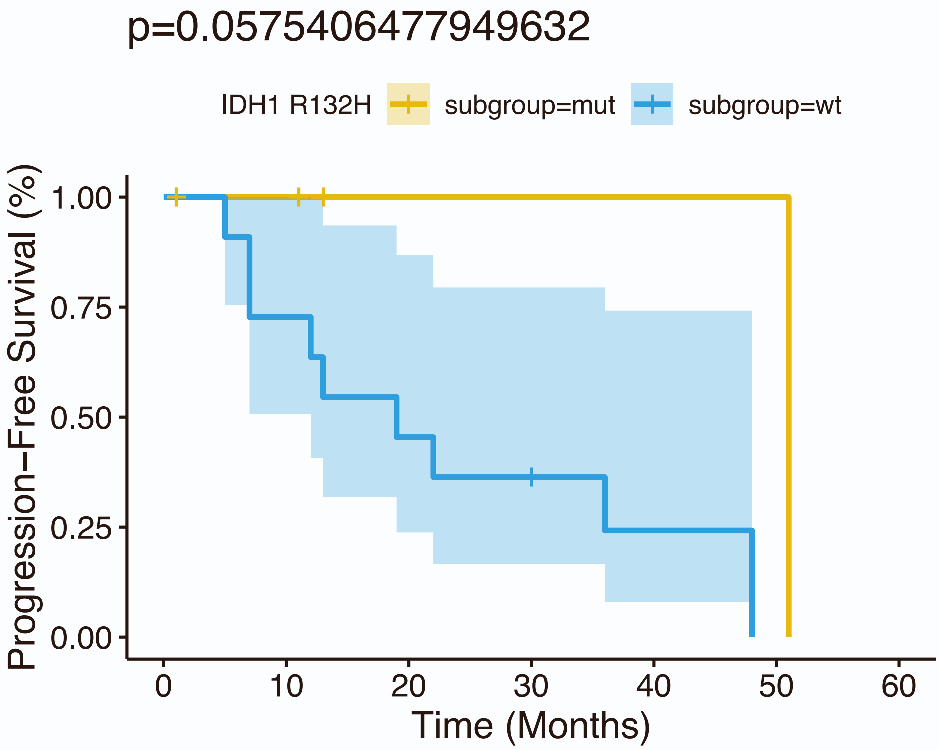

Supplementary. Fig. 3. Kaplan–Meier curves of PFS comparing the IDH-wildtype and IDH-mutant cases, Related to Figure 1.

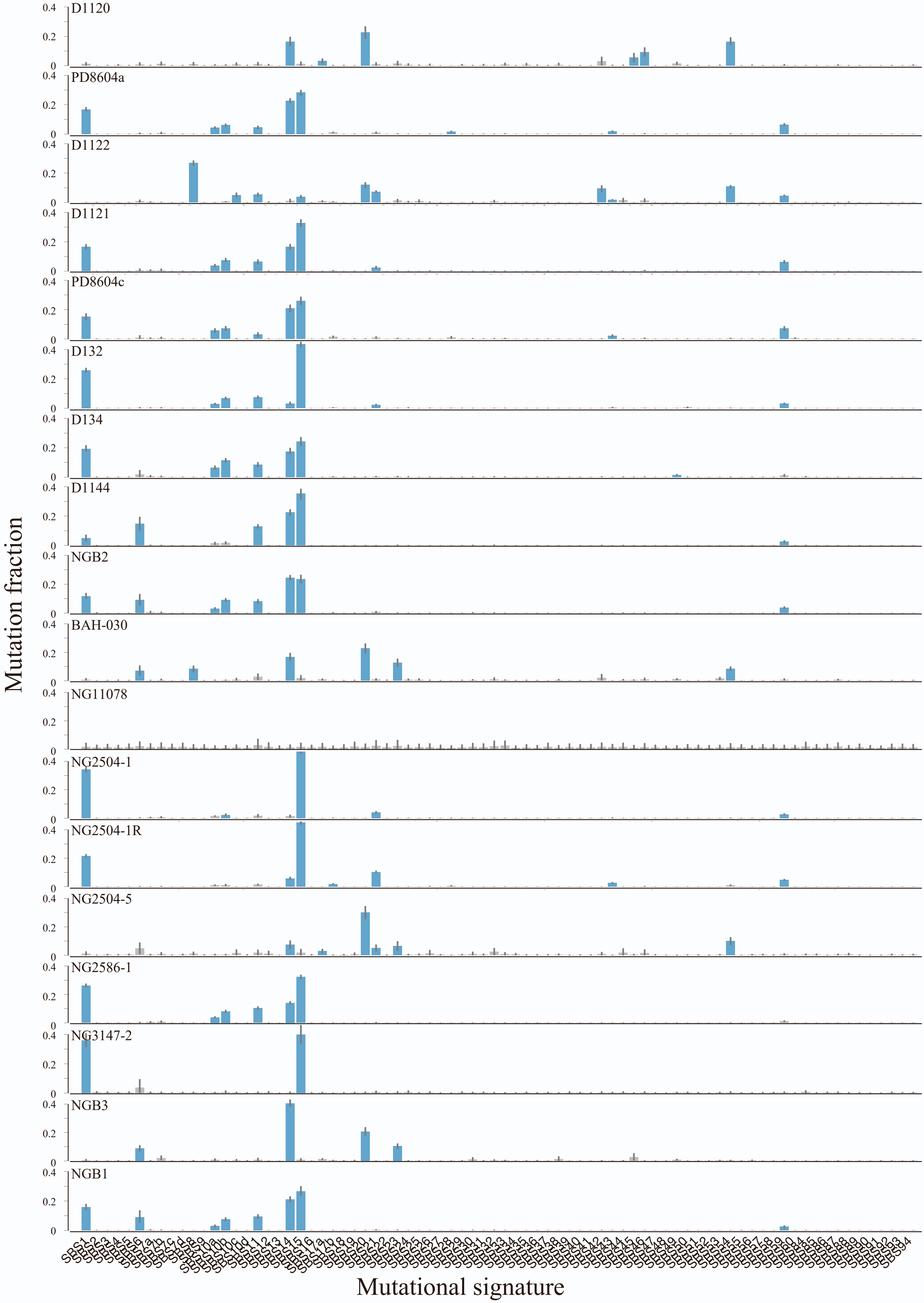

Supplementary. Fig. 4. Mutational signatures in 18 CMMRD HGGs underwent WES.

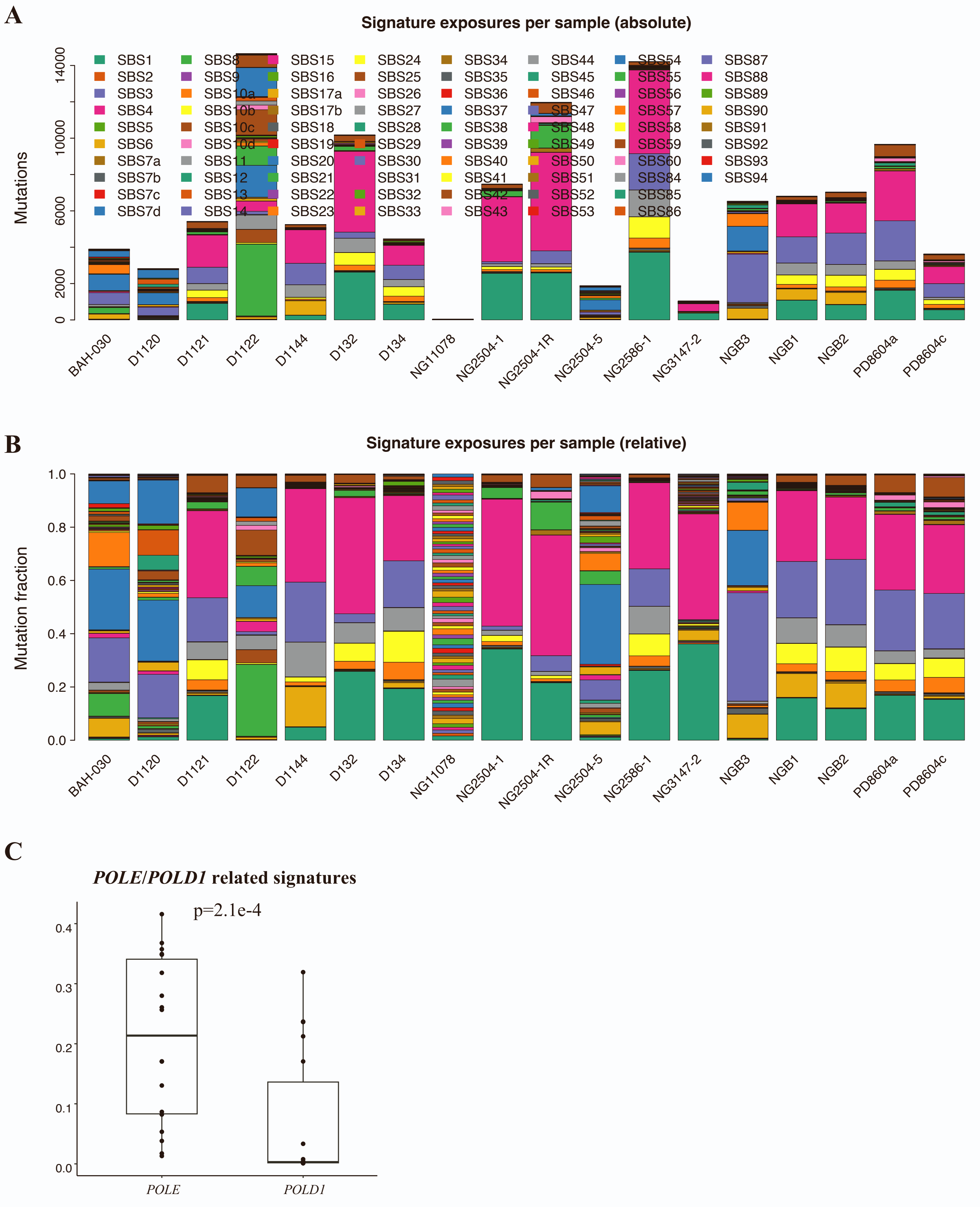

Supplementary. Fig. 5. Summary of mutational signatures in 18 CMMRD HGGs underwent WES, Related to Figure 2. Data are presented as box plots showing the median (center line), interquartile range (box), and outliers (points). * $p < 0.05$, ** $p < 0.01$, *** $p < 0.001$

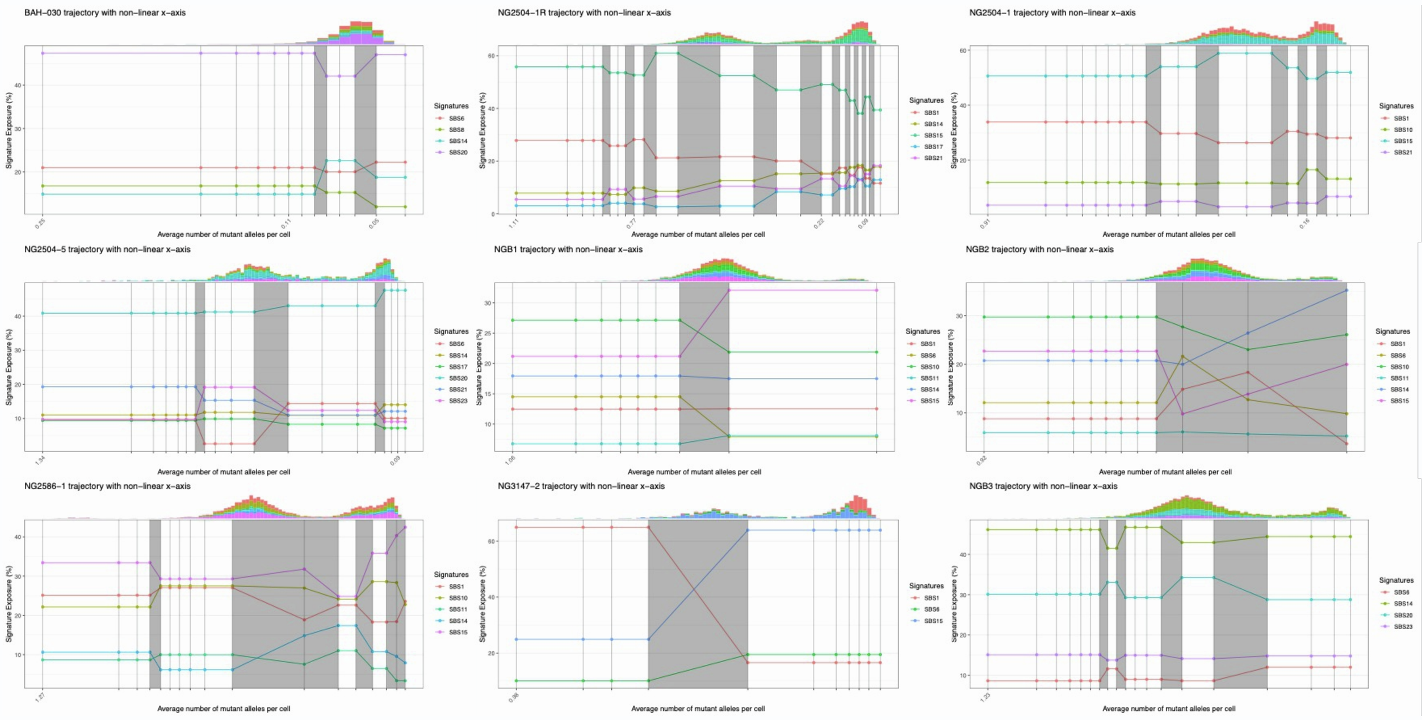

Supplementary. Fig. 6. Changing of mutational signatures over time during clonal evolution, Related to Figure 4.
